# ucOCN Promotes Testosterone Synthesis via the PKA-MAPK/ERK-CREB Signaling Pathway in Porcine Leydig Cells

**DOI:** 10.3390/cells14241937

**Published:** 2025-12-05

**Authors:** Guang Yang, Han Liu, Zhibao Yin, Lihua Zhao, Yanglin Chen, Yiqing Li, Linxin Cheng, Junjun Ma, Jinbo Yu, Yu Zhang, Xihe Li, Rongfeng Li

**Affiliations:** 1 Jiangsu Provincial Key Laboratory of Biological Therapy for Organ Failure, Nanjing Medical University, Nanjing 211166, China; 2 State Key Laboratory of Reproductive Medicine and Offspring Health, Nanjing Medical University, Nanjing 211166, China; 3 Key Laboratory of Targeted Intervention of Cardiovascular Disease, Collaborative Innovation Center for Cardiovascular Disease Translational Medicine, Nanjing Medical University, Nanjing 211166, China; 4 The State Key Laboratory of Reproductive Regulation and Breeding of Grassland Livestock, College of Life Science, Inner Mongolia University, Hohhot 010020, China; 5 National Center of Technology Innovation for Dairy, Hohhot 010020, China; 6 Inner Mongolia Saikexing Institute of Breeding and Reproductive Biotechnology in Domestic Animal, Hohhot 011517, China

**Keywords:** porcine Leydig cells, ucOCN, MEK S298 phospho-switch, PKA-MAPK/ERK-CREB signaling pathway, steroidogenic gene promoter activation

## Abstract

Bone health might be closely associated with male fertility, yet the molecular pathways remain poorly characterized. We demonstrate that undercarboxylated osteocalcin (ucOCN), a bone-derived hormone, initiates a signaling cascade that stimulates testosterone biosynthesis in porcine Leydig cells. Mechanistically, ucOCN binding to membrane receptor GPRC6A elevates intracellular cAMP levels and sequentially activates PKA, MEK, and ERK. ERK translocates to the nucleus and phosphorylates the transcription factor CREB. Activated CREB binds directly to promoter regions of the key steroidogenic genes and boosts testosterone production. The genetic or pharmacological inhibition of GPRC6A, PKA, MEK, or ERK signaling disrupts CREB activation and abolishes both steroidogenic gene expression and testosterone synthesis. Crucially, the phospho-switch S298 as a previously unrecognized phosphorylation site through which MEK regulates osteocalcin (OCN) signaling was identified. Collectively, our results indicate that ucOCN interacts with GPRC6A to promote testosterone synthesis in Leydig cells via the PKA-MAPK/ERK-CREB pathway. The above findings elucidate a fundamental endocrine axis between bone and the male reproductive system, offering novel mechanistic insights and potential therapeutic strategies for improving male fertility.

## 1. Introduction

The skeleton has been traditionally recognized for its roles in support, movement, mineral storage, hematopoiesis, and organ protection. However, recent studies have revealed that bone might function as an endocrine organ, playing a critical role in regulating cognitive functions and energy metabolism [1]. OCN, the main factor secreted by bones, is a non-collagenous bone matrix protein with high conservation across species [2]. It is synthesized in osteoblasts. Most of the synthesized OCN undergoes gamma-carboxylation followed by binding calcium ions and then staying in the bone matrix. The remaining uncarboxylated or partially carboxylated OCN is released into the bloodstream, acting as a multifunctional hormone and being involved in endocrine regulation [3]. Thus, bone emerges not merely as a structural tissue but as an indispensable regulator of systemic homeostasis, underscoring its vital role in human health [4].

It is well known that sex hormones in mammals regulate bone metabolism, such as estrogen modulating female bone health [5], and declining estrogen levels in elderly women increasing osteoporosis risk [6]. Recent clinical data suggest that skeletal health might also be closely linked to male reproductive capability [7,8]. The ucOCN may be the critical regulator of male reproduction [9,10]. Oury group firstly constructed *OCN* gene knockout mice in 2011 and found the *OCN*^−/−^ mice have been associated with significant testicular atrophy, reduced testosterone secretion, decreased sperm count, and impaired male fertility [11,12]. However, another group reported the converse phenomenon that specific deletion of *OCN* in osteoblasts does not affect testosterone synthesis or spermatogenesis [13]. These conflicting findings illustrate the ongoing debate regarding the role of OCN in male reproductive regulation, based on mouse models.

G protein-coupled receptor class C group 6 member A (GPRC6A) is a widely expressed receptor that binds various ligands to exert its functions in tissues including the brain, kidney, skeletal muscle, and testis [14]. Pi et al. demonstrated that GPRC6A serves as the receptor for ucOCN on mouse pancreatic β cells [15,16]. Studies in mice have shown that male mice lacking GPRC6A in the testes display significant reductions in testicular volume, epididymal and seminal vesicle weights, sperm count, and Leydig cell numbers, along with decreased expression levels of genes involved in testosterone synthesis, suggesting that ucOCN may regulate testosterone production in the testes via GPRC6A [12]. Clinical data in men revealed a positive correlation of serum ucOCN levels with testosterone concentrations. Furthermore, the rs224791 polymorphism in the putative receptor GPRC6A gene significantly affects both testosterone levels and spermatogenesis. These findings underscore the important role of the ucOCN-GPRC6A axis in regulating human testicular function [17].

In the canonical G-protein-coupled receptor pathway, ligand binding to GPRC6A catalyzes GDP–GTP exchange on the Gα subunit, yielding active Gα-GTP. This complex then stimulates adenylyl cyclase, thereby increasing intracellular cAMP concentration. The binding of cAMP to the regulatory subunits of protein kinase A (PKA) releases its catalytic subunits, enabling PKA-mediated phosphorylation of CREB [18,19]. However, CREB phosphorylation is not exclusive to PKA; it is also a target of ERK, as demonstrated in murine adipocytes [20,21], myocytes [22], and osteoclast precursors [23]. Critical gaps in knowledge exist regarding how ucOCN-GPRC6A signaling is transmitted within Leydig cells to regulate testosterone production. Key unresolved questions include the following: Do the PKA or MAPK/ERK pathways mediate signaling from ucOCN–GPRC6A to CREB? Which kinase undergoes nuclear translocation to phosphorylate CREB? Does activated CREB directly bind to steroidogenic gene promoters?

The persistent controversy from mouse models and the unclear molecular mechanism necessitate the use of an animal model that more faithfully recapitulates human physiology. Pigs represent an excellent model for this purpose due to their closer genetic, anatomical, and physiological similarities to humans, particularly in reproductive endocrinology [24,25]. Therefore, to resolve the existing controversies and definitively elucidate the role and mechanism of the ucOCN-GPRC6A pathway in male reproduction, we employed the porcine model.

In this study, we aimed to investigate whether and how the ucOCN-GPRC6A axis regulates testosterone biosynthesis in porcine Leydig cells. We hypothesized that ucOCN activates GPRC6A to trigger both the PKA and MAPK/ERK pathways, leading to CREB phosphorylation and subsequent transactivation of steroidogenic gene promoters. Our work seeks to provide a coherent molecular mechanism and deliver more reliable, translatable insights into the bone-testis axis, with implications for further understanding the male infertility.

## 2. Materials and Methods

### 2.1. Isolation of Porcine Leydig Cells

Testes were obtained from 21-day-old piglets, cleaned, disinfected, and transported to the laboratory under cold chain conditions. Under aseptic conditions, the tunica albuginea and fascia were removed. The testicular parenchyma was then stripped and cut into 1–2 mm^3^ pieces. These tissue fragments were digested with a digestive solution (22 mg collagenase I + 1.1 mL FBS + 18.9 mL DMEM/F12) at 37 °C for 45 min with stirring. The primary cell suspension was collected and filtered through a 200-mesh sieve. After time-graded gravitational settling, the supernatant was collected by centrifugation, and the settled tissue mass was subjected to repeated digestion. The resulting supernatants were pooled in DMEM/F12 basal medium. The cells were then purified using gradient Percoll centrifugation, with layers of 100%, 60%, 34%, 26%, and 21% Percoll solutions. Porcine Leydig cells located between the 34% and 60% Percoll layers were collected and cultured in DMEM/F12 medium supplemented with 10% FBS, maintained at 34 °C in a 5% CO_2_ incubator.

### 2.2. Immunofluorescence Assay

The cell suspension was washed twice with DPBS (5 min per wash). Cells were fixed with 4% paraformaldehyde for 10 min at room temperature and then washed three times with DPBS. Permeabilization was performed by incubation with 1% Triton X-100 (in DPBS) for 10 min at room temperature, followed by three DPBS washes. Subsequently, cells were blocked with 10% goat serum for 1 h at room temperature. After blocking, cells were incubated overnight at 4 °C with the primary antibody diluted 1:100 in blocking buffer. The following day, cells were washed three times with DPBS to remove the primary antibody and then incubated with the corresponding secondary antibody for 1 h at room temperature in the dark. After two additional DPBS washes (in the dark), the nuclei were counterstained with Fluoroshield™ mounting medium containing DAPI. Finally, the samples were imaged using a laser confocal microscope. The antibodies are listed in the Supplementary Materials, Table S1.

### 2.3. Co-IP

Porcine Leydig cells in stable passaging culture were treated with 100 ng/mL ucOCN for 24 h. Subsequently, the cells were lysed on ice using NP-40 lysis buffer (containing 50 mM Tris pH 7.4, 0.5% NP-40, 0.01% SDS, and protease inhibitors). The supernatant was collected following centrifugation. The protein concentration of the lysate was determined using the BCA method. An aliquot of the supernatant containing 1 mg of total protein was allocated for the immunoprecipitation (IP) group and the IgG control group, respectively. The remaining lysate was designated as the Input group. For the IP and IgG groups, the lysates were incubated with the specific IP antibody or control IgG antibody overnight at 4 °C with gentle agitation. The following day, Protein A/G beads, which had been pre-washed with NP-40 lysis buffer, were added to each mixture and incubated for an additional 6 h at 4 °C. After incubation, the beads were collected using a magnetic rack and washed. The bound proteins were then eluted and denatured by boiling in 2× Loading Buffer at 95 °C for 10 min. Finally, the eluted proteins (from the IP and IgG groups) and the Input lysate were subjected to Western blot analysis.

### 2.4. Testosterone and cAMP Measurements

To eliminate potential interference from exogenous hormones and growth factors present in serum, the culture medium was replaced with a defined, serum-free DMEM/F12 medium supplemented with 0.1% (*w*/*v*) bovine serum albumin (BSA) prior to all experimental treatments. The cells were then stimulated under these optimized conditions for the indicated times to ensure that measured changes in testosterone and cAMP levels were attributable solely to the experimental stimuli.

Cell supernatants were collected, centrifuged (1000× *g*, 10 min, 4 °C) to remove debris, and stored at −80 °C until analysis. Concentrations of cAMP and testosterone were determined using commercial competitive and sandwich ELISA kits, respectively (Cat. AF084049-A and AF04562-A; aifang biological, Changsha, China), strictly following the manufacturers’ instructions. Briefly, 50 μL of standard or 10 μL of sample plus 40 μL of Sample Diluent were added per well. Subsequently, 50 μL of HRP-conjugated competing antigen (cAMP) or 100 μL of HRP-conjugated detection antibody (testosterone) was introduced. After 60 min incubation at 37 °C, plates were washed five times, developed with 50 μL each of Substrate A and B for 15 min at 37 °C in the dark, and stopped with 50 μL of Stop Solution. Optical density was read at 450 nm within 15 min. Values were interpolated from a four-parameter logistic standard curve and multiplied by the dilution factor (×5) to obtain the actual analyte concentration in the supernatant. The lower limit of detection was 1.0 pg /mL for both assays; intra- and inter-assay coefficients of variation were <10%.

### 2.5. Western Blot

Under low-temperature conditions, tissues were homogenized using RIPA lysis buffer, while cells were lysed with NP-40 lysis buffer containing a protease inhibitor. The lysates were then centrifuged to collect the supernatant. After determining the protein concentration by the BCA method, 20–40 µg of the supernatant protein was separated by SDS-PAGE and transferred to a PVDF membrane. The membrane was blocked with 5% skim milk for 2 h, followed by incubation with the primary antibody at 4 °C overnight and the corresponding secondary antibody at room temperature for 1 h. After washing three times with TBST, the membrane was developed and exposed using an ECL luminescence solution. The antibodies are listed in the Supplementary Materials, Table S1.

### 2.6. CUT&Tag

Following the instructions of the Novong CUT&Tag high-sensitivity kit (NovoProtein, Shanghai, China), we performed CUT&Tag library amplification and purification: Add 100 ng/mL ucOCN to the stable-adapted porcine Leydig cell culture system, incubate for 24 h, then collect samples. Count 50,000 cells and fix them onto Bean protein A magnetic beads to permeabilize. Resuspend the cells in antibody buffer, then sequentially incubate with primary antibody against CREB and secondary goat anti-rabbit IgG H&L antibody to allow antibody entry and binding to the target protein. Next, introduce pAG-Tn5 transposome, which binds to the antibody and is fixed onto the target protein. Activate Tn5 enzyme with Mg^2+^ to cleave the DNA regions bound by the target protein. After fragmentation, extract the DNA, then perform amplification and purification to construct the library. Sequence the purified DNA library on the personalbio platform to a depth of 6G per sample. Use IGV v2.14.1 for data visualization. This experiment verifies the binding of CREB to the promoter regions of key genes involved in testosterone synthesis in porcine Leydig cells.

### 2.7. Nucleus-Cytoplasm Fractionation of Porcine Leydig Cells

Porcine Leydig cells were subjected to nuclear and cytoplasmic separation using the Cell Fractionation Kit (SC-003 Minute™, Invent Biotechnologies, Peking, China) following the manufacturer’s instructions. The separated nuclear and cytoplasmic proteins were then extracted and analyzed by Western blot. The expression levels of p-ERK1/2 were detected in each fraction. GAPDH served as the cytoplasmic protein control, while H3 histone was used as the nuclear protein control. Protein quantification was performed using ImageJ 1.48u software (Bio-Rad, Hercules, CA, USA). The relative expression of p-CREB was quantified by calculating the grayscale intensity ratio of p-CREB to each control protein.

### 2.8. siRNA and Transfection

GPRC6A siRNA and CREB siRNA were synthesized by RIBOBIO (Guangzhou, China). The siRNA transfection was performed according to the manufacturer’s protocol using siRNA Transfection Reagents (Shanghai Biotend Biotechnology Co., Ltd; Shanghai, China).

### 2.9. Overexpression Plasmid Transfection

The CREB overexpression plasmid was purchased from Sangon (Shanghai, China). The plasmid transfection was carried out following the manufacturer’s instructions using Liposomal nucleic acid transfection reagent (Yeasen Biotechnology, Shanghai, China).

### 2.10. Chemical Treatments

Porcine testicular Leydig cells were treated with recombinant porcine osteocalcin fused with a GST-tag (QCR471Po01, QCHENG BIO, Shanghai, China). The following pharmacological inhibitors were also used: the MEK inhibitor trametinib (T2125, TargetMol, Boston, MA, USA), the PKA inhibitor H-89 (T11524, TargetMol, Boston, MA, USA), the ERK inhibitor SCH772984 (T6066, TargetMol, Boston, MA, USA), and staurosporine (T6680, TargetMol, Boston, MA, USA).

### 2.11. RT-QPCR

Total RNA was extracted from porcine Leydig cells using the Cellular RNA Extraction Kit. Subsequently, the RNA was reverse transcribed into cDNA using the HiScript II Q RT Super-Mix for qPCR Reverse Transcription Kit (Vazyme, Nanjing, China). The expression levels of the target genes were quantified using the ChamQ SYBR qPCR Master Mix Kit (Vazyme, Nanjing, China) with gene-specific primers on a real-time PCR system. GAPDH was used as the reference gene for normalization. RT-QPCR primer sequences are listed in Supplementary Materials, Table S2.

### 2.12. Statistical Analysis

All data in this experiment were expressed as mean ± standard deviation (X ± S) and GraphPad Prism9 was used for significance analysis. A t-test was used to compare the two independent samples; one-way ANOVA was used to compare multiple samples and two-way ANOVA (or mixed model) was used to compare multiple groups and *p* ≤ 0.05 was considered statistically significant. In all graphs, *p* ≤ 0.05 was marked as *, *p* ≤ 0.01 was marked as **, *p* ≤ 0.001 was marked as ***, *p* ≤ 0.0001 was marked as ****, and *n* ≥ 3; ns, not significant.

## 3. Results

### 3.1. High Similarity of OCN Between Pigs and Humans

To assess how similar OCN from mice, pigs, and humans is, we compared their amino acid sequences using data from GenBank. The results showed that porcine and human OCN are about 86% identical, while murine and human OCN share only approximately 60% (Figure S1A,B). Structural analysis of secondary structures found that both human and porcine OCN consist of roughly 61% alpha-helices, whereas murine OCN has a slightly higher proportion at about 65.3%. The amounts of irregular coils are 37% in human, 35% in pig, and 27.4% in mouse (Figure S1C). These observations suggest that human and porcine OCN are more conserved in their secondary structural features. Additionally, 3D modeling and comparison using PyMOL (v3.0.3)yielded root mean square deviation (RMSD) values of 0.0027 between human and pig, and 0.003 between human and mouse, indicating that human and porcine OCN share very similar tertiary structures (Figure S1D). Based on these findings, we conclude that porcine OCN is more functionally similar to human OCN compared to mouse, supporting the idea that pigs are a more relevant model for studying OCN-related physiology.

### 3.2. Isolation and Identification of Porcine Leydig Cells

**Figure 1:**
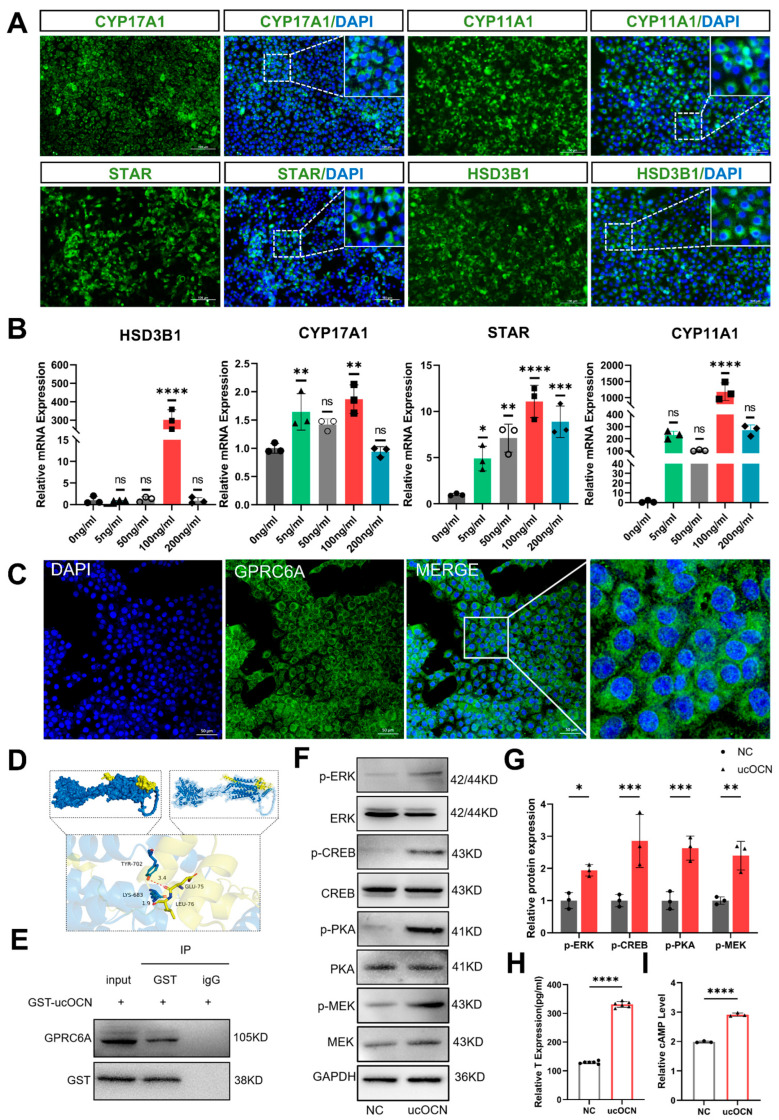
The exogenous ucOCN promotes the expression of genes encoding enzymes necessary for testosterone biosynthesis. GPRC6A serves as the receptor of ucOCN in porcine Leydig cells. The downstream kinases of GPRC6A and their specific phosphorylation sites. (**A**) Immunofluorescence detection of the expression of the key enzymes required for testosterone synthesis HSD3B1, STAR, CYP11A1, and CYP17A1 in porcine Leydig cells (Scale bars = 100 µm); the dashed box areas are magnified. (**B**) RT-PCR analysis of gene expression levels of four key enzymes required for testosterone synthesis (including *HSD3B1*) in porcine Leydig cells treated with different concentrations of ucOCN (5, 50, 100, 200 ng/mL) (*n* = 3 biological replicates). (**C**) Immunofluorescence confirmation of GPRC6A expression on the membrane of porcine Leydig cells (Scale bars = 50 µm). (**D**) Molecular docking prediction of the interaction between GPRC6A and OCN. (**E**) Co-immunoprecipitation (Co-IP) analysis confirming the interaction between GPRC6A and ucOCN in porcine Leydig cells treated with 100 ng/mL ucOCN. (**F**) Western blot analysis of total and phosphorylated protein expression levels of PKA, MEK, ERK, and CREB in porcine Leydig cells before and after treatment with 100 ng/mL exogenous ucOCN (*n* = 3 biological replicates). (**G**) Quantification of the Western blot results shown in (**F**). (**H**) ELISA detection of testosterone levels in porcine Leydig cells before and after treatment with 100 ng/mL exogenous ucOCN (*n* = 6 biological replicates). (**I**) ELISA detection of cAMP levels in porcine Leydig cells before and after treatment with 100 ng/mL exogenous ucOCN (*n* = 3 biological replicates). Data are represented as means ± SD. * *p* < 0.05, ** *p* < 0.01, *** *p* < 0.001, **** *p* < 0.0001; ns, not significant.

Primary porcine Leydig cells were isolated via Percoll gradient centrifugation and cultured in vitro (Figure S2A). Subsequent characterization included analysis of key testosterone synthesis genes including *HSD3B1*, *CYP11A1*, *CYP17A1*, *STAR*, and the Leydig cell marker *INSL3* using RT-PCR. The results indicated that these isolated cells consistently expressed these enzyme genes and *INSL3* (Figure S2B). Immunofluorescence assays for HSD3B1, CYP11A1, CYP17A1, and STAR further validated their identity as Leydig cells (Figure 1A). Collectively, these cultured cells provide a reliable model to investigate the molecular mechanisms underlying OCN’s effects on Leydig cell function in vitro.

### 3.3. The Exogenous ucOCN Promotes the Expression of Genes Encoding Enzymes Necessary for Testosterone Biosynthesis

The process of testosterone synthesis in Leydig cells is regulated by multiple enzymes, among which *STAR*, *HSD3B1*, *CYP11A1*, and *CYP17A1* are key enzymes and serve as markers to assess the cells’ capacity for testosterone production. Previous studies reported that serum OCN concentrations in pigs range from 28.5 to 188.3 ng/mL, with an average concentration of 90.2 ng/mL [26]. Based on this, we added different concentrations of ucOCN (0, 5, 50, 100, 200 ng/mL) to the culture medium of porcine Leydig cells. After 24 h of treatment, we employed RT-qPCR to detect the expression levels of key testosterone synthesis enzyme genes (*STAR*, *HSD3B1*, *CYP11A1*, *CYP17A1*). The results showed that exogenous ucOCN significantly promoted the expression of these genes in porcine Leydig cells, with the most pronounced effect observed at 100 ng/mL (Figure 1B).

### 3.4. GPRC6A Serves as the Receptor of ucOCN in Porcine Leydig Cells

To determine whether GPRC6A is expressed in porcine Leydig cells, we performed immunofluorescence staining. This analysis revealed the specific expression of GPRC6A on the plasma membrane of porcine Leydig cells (Figure 1C). To explore whether GPRC6A functions as the receptor for ucOCN, we utilized the STRING database and molecular docking predictions. The results suggested an interaction between ucOCN and GPRC6A Figure 1D and Figure S2C). To validate this interaction experimentally, we added 100 ng/mL ucOCN to cultured porcine Leydig cells and performed co-immunoprecipitation (Co-IP). Results confirmed that GPRC6A physically interacts with ucOCN in porcine Leydig cells (Figure 1E).

### 3.5. The Downstream Kinases of GPRC6A and Their Specific Phosphorylation Sites

The exogenous ucOCN treatment elevated cAMP levels, leading to the phosphorylation of PKA and the activation of downstream signaling molecules including MEK, ERK and CREB. Specifically, PKA was activated via phosphorylation at T197, MEK was phosphorylated at S298 and S218/S222, ERK1/ERK2 were phosphorylated at T202/Y204 and T185/Y187, respectively. The transcription factor CREB was also phosphorylated at S133 (Figure 1F,G). These signaling events collectively promoted testosterone and cAMP synthesis (Figure 1H,I). Collectively, these results confirm that ucOCN facilitates downstream signaling activation, ultimately promoting testosterone biosynthesis and secretion in Leydig cells.

### 3.6. Confirmation of GPRC6A Role in ucOCN-Mediated CREB Phosphorylation and Testosterone Synthesis Signal Pathway

**Figure 2:**
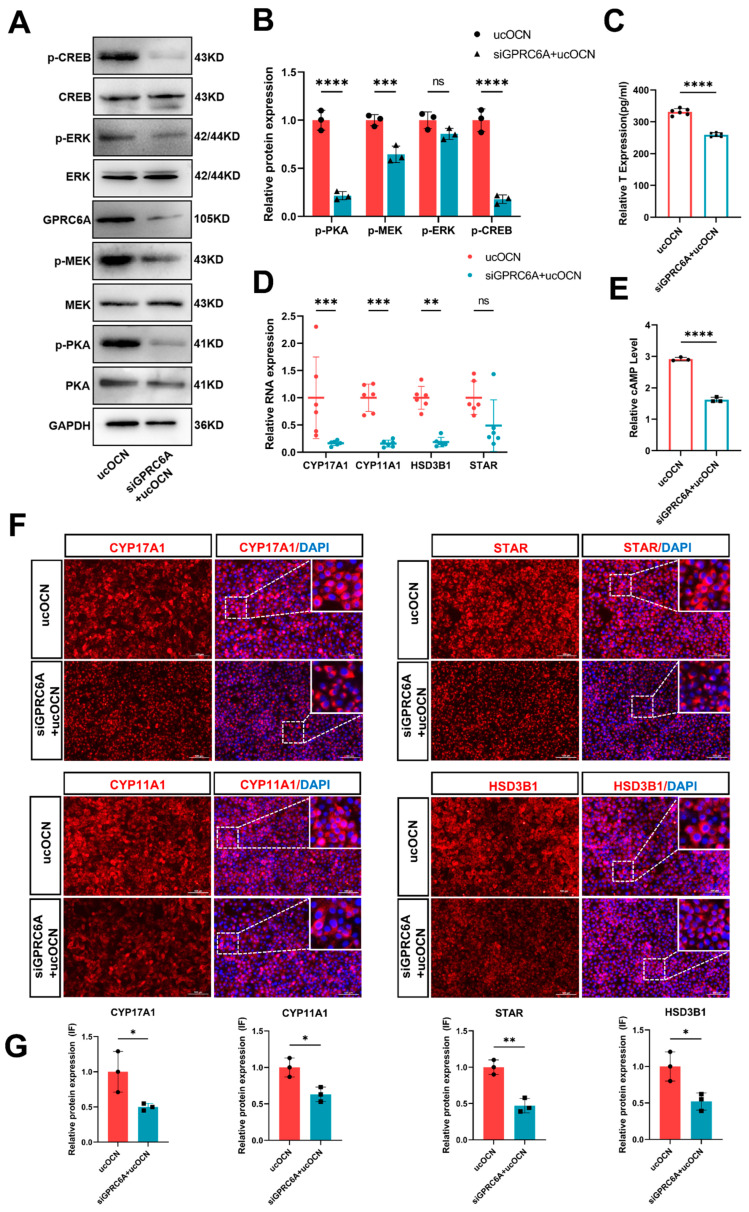
Confirmation of GPRC6A role in ucOCN-mediated CREB phosphorylation and testosterone synthesis signal pathway. (**A**) Western blot analysis of total and phosphorylated protein expression levels of PKA, MEK, ERK, and CREB in porcine Leydig cells treated with 100 ng/mL ucOCN, with or without GPRC6A knockdown (*n* = 3 biological replicates). (**B**) Quantification of the Western blot results shown in (**A**). (**C**) ELISA detection of testosterone levels in porcine Leydig cells treated with 100 ng/mL ucOCN, with or without GPRC6A knockdown (*n* = 6 biological replicates). (**D**) RT-qPCR analysis of gene expression levels of key enzymes required for testosterone synthesis (*STAR*, *HSD3B1*, *CYP17A1*, *CYP11A1*) in porcine Leydig cells treated with 100 ng/mL ucOCN, with or without GPRC6A knockdown (*n* = 6 biological replicates). (**E**) ELISA detection of cAMP levels in porcine Leydig cells treated with 100 ng/mL ucOCN, with or without GPRC6A knockdown (*n* = 3 biological replicates). (**F**) Immunofluorescence (IF) detection of the protein expression levels of key enzymes required for testosterone synthesis (STAR, HSD3B1, CYP11A1, and CYP17A1) in porcine Leydig cells treated with 100 ng/mL ucOCN, with or without GPRC6A knockdown (*n* = 3 biological replicates) (Scale bars = 100 µm); the dashed box areas are magnified. (**G**) Quantification of the immunofluorescence (IF) intensity shown in (**F**). Data are represented as means ± SD. * *p* < 0.05, ** *p* < 0.01, *** *p* < 0.001, **** *p* < 0.0001; ns, not significant.

To further elucidate the role of the ucOCN receptor GPRC6A in the pathway leading to CREB activation and increased testosterone production, we designed siRNA targeting GPRC6A. porcine Leydig cells were transfected with 60 nM siGPRC6A to knock down GPRC6A expression, followed by treatment with 100 ng/mL ucOCN for 24 h. Western blot analysis revealed that GPRC6A knockdown significantly reduced the phosphorylation of key signaling molecules PKA, MEK, ERK1/2, and CREB, concomitant with a decrease in testosterone production (Figure 2A–C). RT-qPCR demonstrated that GPRC6A inhibition downregulated the expression of key enzyme genes of testosterone synthesis, including *STAR, HSD3B1*, *CYP17A1*, and *CYP11A1*. Notably, ucOCN treatment failed to rescue the levels of these enzymes (Figure 2D). Furthermore, measurement of cAMP levels indicated that GPRC6A silencing attenuated cAMP production (Figure 2E). Similarly, immunofluorescence analysis confirmed reduced expression of key enzymes required for testosterone synthesis STAR, HSD3B1, CYP17A1, and CYP11A1 following GPRC6A knockdown (Figure 2F,G). Collectively, these results demonstrate the critical role of GPRC6A in this signaling cascade and its impact on downstream molecules controlling testosterone synthesis.

### 3.7. ucOCN Promotes Testosterone Synthesis via the PKA-MAPK/ERK Signaling Pathway

**Figure 3:**
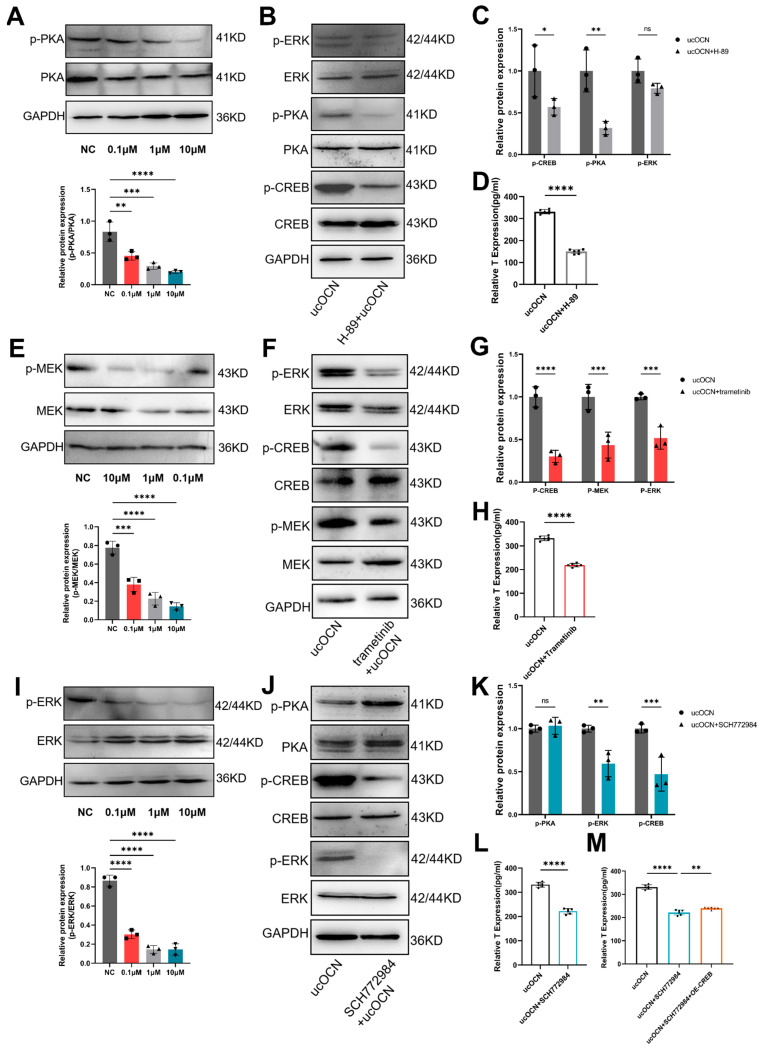
Osteocalcin promotes testosterone synthesis via the PKA–MAPK/ERK signaling pathway in porcine Leydig cells. (**A**) Western blot analysis of total and phosphorylated PKA protein levels in cells treated with 100 ng/mL ucOCN together with increasing concentrations (0.1, 1, 10 µM) of the PKA inhibitor H-89. (**B**,**C**) Western blot analysis and quantification of total and phosphorylated levels of PKA, ERK, and CREB in cells treated with 100 ng/mL, with or without 10 µM H-89. (**D**) Testosterone levels measured by ELISA in cells treated with 100 ng/mL ucOCN, with or without 10 µM H-89. (**E**) Western blot analysis of total and phosphorylated MEK protein levels in cells treated with 100 ng/mL ucOCN together with increasing concentrations (0.1, 1, 10 µM) of the MEK inhibitor Trametinib. (**F**,**G**) Western blot analysis and quantification of total and phosphorylated levels of MEK, ERK, and CREB in cells treated with 100 ng/mL ucOCN, with or without 10 µM Trametinib. (**H**) Testosterone levels measured by ELISA in cells treated with 100 ng/mL ucOCN, with or without 10 µM Trametinib. (**I**) Western blot analysis of total and phosphorylated ERK protein levels in cells treated with 100 ng/mL ucOCN together with increasing concentrations (0.1, 1, 10 µM) of the ERK inhibitor SCH772984. (**J**,**K**) Western blot analysis and quantification of total and phosphorylated levels of PKA, ERK, and CREB in cells treated with 100 ng/mL ucOCN, with or without 1 µM SCH772984. (**L**) Testosterone levels measured by ELISA in cells treated with 100 ng/mL ucOCN, with or without 1 µM SCH772984. (**M**) Testosterone levels measured under the following treatments: 100 ng/mL ucOCN (control), 100 ng/mL ucOCN plus 1 µM SCH772984, and 100 ng/mL ucOCN plus 1 µM SCH772984 followed by CREB overexpression. Data are presented as mean ± SD (*n* = 3 biological replicates for Western blot; *n* = 6 biological replicates for ELISA). * *p* < 0.05, ** *p* < 0.01, *** *p* < 0.001, **** *p* < 0.0001; ns, not significant.

To investigate the functional order of the involved kinases after ucOCN triggers the cAMP production via binding to GPRC6A, porcine Leydig cells were treated with 100 ng/mL ucOCN and the PKA inhibitor H-89. After 24 h, protein samples were collected. Initial tests identified 10μM as the optimal H-89 concentration (Figure 3A). Western blot results showed that this inhibitor significantly reduced the phosphorylation of PKA, ERK, and CREB in ucOCN-treated cells, along with decreased testosterone secretion (Figure 3B–D). These findings indicate that PKA acts upstream of MEK, ERK, and CREB in this signaling pathway.

To clarify the role and its functional order of MEK in this signaling pathway, porcine Leydig cells treated with 100 ng/mL ucOCN were exposed to the MEK inhibitor trametinib. After 24 h, proteins were analyzed. Preliminary testing identified 10μM as the effective inhibitory dose (Figure 3E). Western blot analysis showed that blocking MEK markedly decreased the phosphorylation of MEK, ERK, and CREB, leading to reduced testosterone synthesis and secretion (Figure 3F–H), indicating that MEK acts upstream of ERK and CREB in this pathway.

To verify ERK’s role and functional order in this pathway, porcine Leydig cells treated with 100 ng/mL ucOCN were exposed to the ERK inhibitor SCH772984. After 24 h, proteins were extracted for analysis. Early experiments determined that 1μM SCH772984 effectively blocked ERK phosphorylation (Figure 3I). Western blot results showed that inhibiting ERK decreased phosphorylation of both ERK and CREB, notably, PKA phosphorylation remained unchanged (Figure 3J,K), and this was associated with reduced testosterone synthesis and secretion (Figure 3L). Importantly, co-overexpression of CREB partially restored testosterone levels despite ERK inhibition (Figure 3M). These results indicate that ERK kinase regulates CREB activity within this pathway. Overall, the data confirm that ucOCN released by osteoblasts influences testosterone production via the PKA-MAPK/ERK-CREB signaling cascade, with ERK functioning as an upstream regulator of CREB.

### 3.8. Phosphorylated ERK (p-ERK) Enters the Nucleus to Activate the Transcription Factor CREB

**Figure 4:**
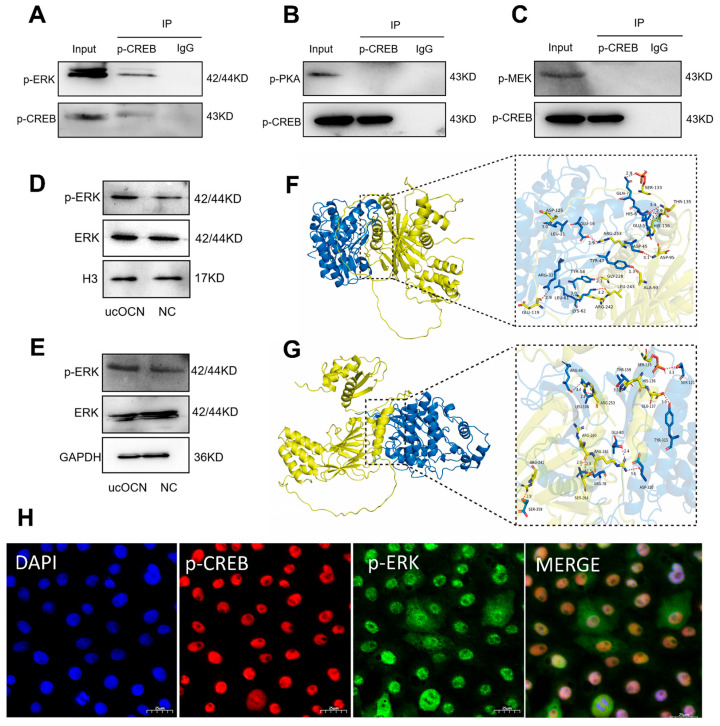
p-ERK enters the nucleus to activate the transcription factor CREB. (**A**) Co-IP analysis confirming the interaction between p-ERK and p-CREB in porcine Leydig cells treated with 100 ng/mL ucOCN. (**B**) Co-IP analysis showing no detectable interaction between p-PKA and p-CREB in porcine Leydig cells treated with 100 ng/mL ucOCN. (**C**) Co-IP analysis showing no detectable interaction between p-MEK and p-CREB in porcine Leydig cells treated with 100 ng/mL ucOCN. (**D**,**E**) Western blot analysis of total and phosphorylated ERK protein levels in nuclear and cytoplasmic fractions extracted from porcine Leydig cells treated with or without 100 ng/mL ucOCN. (**F**) Molecular docking prediction of an interaction between ERK1 and p-CREB. (**G**) Molecular docking prediction of an interaction between ERK2 and p-CREB. (**H**) Immunofluorescence co-localization analysis confirming the interaction between p-CREB and p-ERK (Scale bars = 25 µm).

In our research, we confirmed that ucOCN signals via the PKA-MAPK/ERK pathway. However, it was unclear which kinase in this cascade directly enters the nucleus to activate CREB. To answer this issue, we treated porcine Leydig cells with 100 ng/mL ucOCN. Co-IP assays showed that p-ERK directly binds to phosphorylated CREB (p-CREB) (Figure 4A). In contrast, neither phosphorylated PKA (p-PKA) nor phosphorylated MEK (p-MEK) interacted with p-CREB (Figure 4B,C).

Subsequent nuclear-cytoplasmic fractionation assays showed that some p-ERK moves into the nucleus after activation (Figure 4D,E). To further confirm the interaction between ERK and CREB, molecular docking suggested a possible binding between ERK1/ERK2 and p-CREB (Figure 4F,G). Additionally, fluorescence co-localization experiments verified that p-ERK and p-CREB co-exist within the cell nucleus, supporting a direct interaction (Figure 4H). These results indicate that CREB phosphorylation is directly facilitated by ERK, rather than PKA or MEK. Collectively, our data confirm that activated p-ERK translocates into the nucleus to stimulate CREB activity.

### 3.9. CREB Binds to the Promoter Regions of Genes Encoding Enzymes Necessary for Testosterone Biosynthesis and Transcriptionally Activates Their Expression

To investigate whether the transcription factor CREB is involved in regulating genes critical for testosterone synthesis, we utilized the JASPAR database (jaspar.genereg.net/) (accessed on 5 April 2024) for predictive analysis. The results indicated that CREB can bind to the promoter regions of key testosterone synthesis enzyme genes—including *CYP11A1*, *CYP17A1*, *HSD3B1*, and *STAR*—at the consensus binding motif TGACGTCA (Figure S3A).

**Figure 5:**
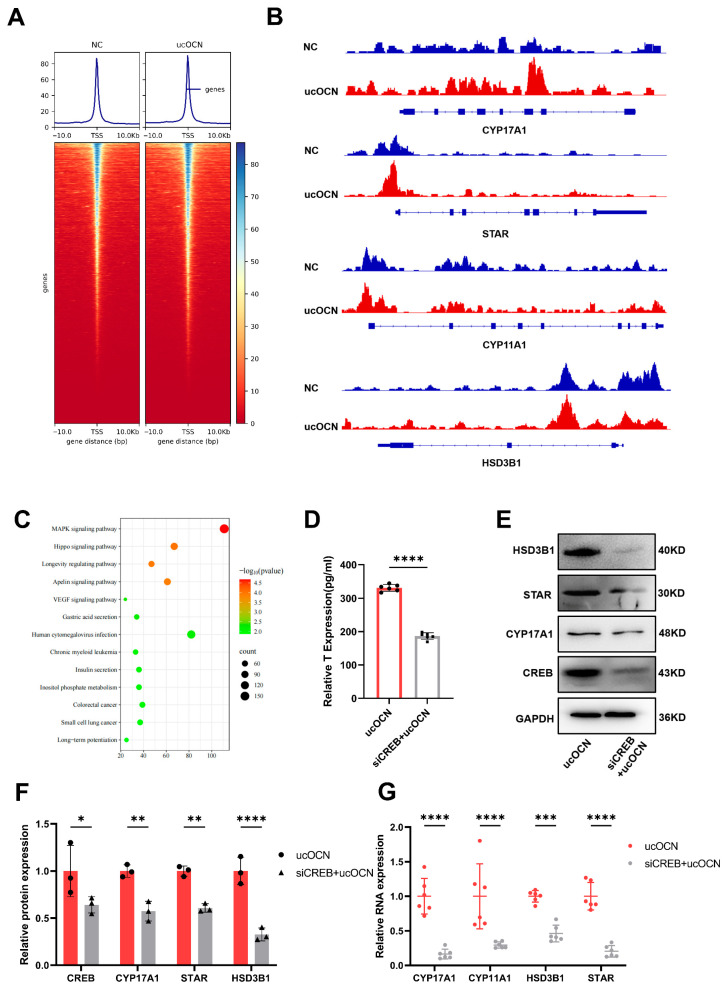
CREB binds to the promoter regions of genes encoding enzymes necessary for testosterone biosynthesis and transcriptionally activates their expression. (**A**,**B**) CUT&TAG analysis demonstrating binding of p-CREB to the genes encoding four key enzymes required for testosterone synthesis (including HSD3B1). The IGV peak heights for these genes were higher in the ucOCN-treated group compared to the negative control (NC) group (*n* = 2 biological replicates). (**C**) KEGG pathway analysis showing significant enrichment of the MAPK signaling pathway. (**D**) ELISA detection of testosterone levels in porcine Leydig cells treated with 100 ng/mL ucOCN, with or without CREB knockdown (*n* = 6 biological replicates). (**E**) Downregulation of CREB and key enzymes required for testosterone synthesis (HSD3B1, STAR, CYP17A1) upon knockdown of the transcription factor CREB (*n* = 3 biological replicates). (**F**) Quantification of the results shown in (**E**). (**G**) RT-qPCR analysis of gene expression levels of key enzymes required for testosterone synthesis (*STAR*, *HSD3B1*, *CYP17A1*, *CYP11A1*) in porcine Leydig cells treated with 100 ng/mL ucOCN, with or without CREB knockdown (*n* = 6 biological replicates). Data are represented as means ± SD. * *p* < 0.05, ** *p* < 0.01, *** *p* < 0.001, **** *p* < 0.0001.

To validate CREB’s regulatory function, we used CUT&Tag technology, confirming target genes regulated by CREB. Our data showed that p-CREB binds to the promoters of *STAR*, *HSD3B1*, *CYP11A1*, and *CYP17A1* in porcine Leydig cells. Importantly, treatment with 100 ng/mL ucOCN enhanced CREB’s binding to these promoters and increased their expression levels, which are key enzymes in testosterone biosynthesis (Figure 5A,B). KEGG pathway analysis revealed significant enrichment of the MAPK/ERK pathway, aligning with earlier findings (Figure 5C).

Furthermore, CREB knockdown reduced its occupancy at the promoters of key steroidogenic genes and reduced testosterone secretion; this effect was not rescued by exogenous ucOCN (Figure 5D and Figure S3B,C). Correspondingly, CREB knockdown or inhibition downregulated the expression of key enzymes (such as STAR) at both the protein and mRNA levels (Figure 5E–G). Collectively, these findings indicate that ucOCN-induced CREB phosphorylation stimulates testosterone production by directly binding to and transactivating key genes in the steroidogenic pathway.

### 3.10. ucOCN Exhibits No Adverse Effects on Porcine Leydig Cells and Porcine Vascular Endothelial Cells

To investigate whether ucOCN has any adverse effects, we employed porcine Leydig cells and porcine vascular endothelial cells (PVEC) as model systems. Immunofluorescence staining of primary porcine vascular endothelial cells confirmed the expression of the endothelial cell-specific marker CD31 (Figure S5A). We conducted CCK-8 cell viability and proliferation identification on wild-type porcine Leydig cells and porcine Leydig cells treated with 5 ng/mL, 50 ng/mL, or 100 ng/mL ucOCN, or 1 μM Staurosporine (STS). We found that the addition of ucOCN did not affect the viability and cell proliferation of porcine Leydig cells, and did not produce any side effects. In contrast, STS treatment induced progressive cell death (Figure S4A,B). Subsequently, we performed the CCK-8 assay on PVEC treated with 100 ng/mL ucOCN or 1 µM STS. Consistent with the porcine Leydig cells results, 100 ng/mL ucOCN did not affect PVEC viability or proliferation (Figure S5B,C). These findings indicate that ucOCN treatment across the tested concentrations (5–100 ng/mL) did not affect the viability or proliferation of either cell type, suggesting an absence of cytotoxic effects at these doses. Furthermore, the concentration (100 ng/mL) of ucOCN was effective in significantly promoting the expression of key enzymes involved in testosterone synthesis in porcine Leydig cells, demonstrating its beneficial impact on porcine Leydig cells.

## 4. Discussion

Leydig cells, resident in the testicular interstitium, synthesize over 95% of circulating testosterone in males [27]. As the predominant androgen, testosterone plays a central role in maintaining male reproductive functions [28,29], spermatogenesis, secondary sexual characteristics, musculoskeletal health, metabolic equilibrium, and neurocognitive functions [30,31]. Emerging evidence indicates that its synthesis is modulated by various intrinsic and extrinsic cues, ranging from endogenous metabolites such as β-hydroxybutyrate to exogenous compounds like apigenin and peptides derived from *Trichosanthes kirilowii* [32,33]. However, the ucOCN-mediated endocrine function of bone in male reproduction remains poorly defined [11]. Although an in vitro study on buffalo Leydig cells reported that ucOCN stimulates testosterone production, it failed to verify GPRC6A as the functional receptor and did not elucidate the underlying signaling mechanism [34]. Here, we systematically decipher the signaling mechanism through which ucOCN potentiates testosterone synthesis in porcine Leydig cells, identifying critical regulatory nodes and providing unprecedented mechanistic insight into the bone-testis axis.

Unlike previous reports that primarily described phenotypic deficiencies in GPRC6A knockout mice [11], our work provides direct evidence for the functional interaction between ucOCN and GPRC6A in a porcine model, employing Co-IP and molecular docking. Furthermore, we demonstrated that ucOCN upregulates key steroidogenic enzymes, including HSD3B1, and promotes testosterone synthesis via GPRC6A-mediated CREB activation. Moreover, ucOCN drove a non-monotonic transcriptional response in steroidogenic genes: expression peaked at 100 ng/mL and waned at 200 ng/mL. This classic inverted-U dose–response signature suggests that supra-optimal concentrations may trigger ligand-induced GPRC6A desensitization or compensatory feedback shutdown [35,36,37]. A major finding of our study is the identification of a dual signaling pathway activated by ucOCN–GPRC6A engagement, which comprises the classical cAMP-PKA axis and the MAPK/ERK cascade. Our data establish a sequential PKA-to-MEK/ERK activation axis in Leydig cells. This cascade aligns with established mechanisms of PKA–MAPK crosstalk, wherein PKA directly engages upstream components of the ERK pathway through multiple well-documented routes. These include PKA-mediated phosphorylation and activation of B-Raf, Rap1-dependent potentiation of B-Raf signaling, and Rac–PAK–driven enhancement of MEK activity—notably via phosphorylation at Ser298 [38,39,40]. Our observation that ucOCN–GPRC6A signaling enhances MEK-Ser298 phosphorylation further underscores the relevance of the Rac–PAK–MEK axis in this endocrine context, thereby providing a mechanistic explanation for the integrated regulation of steroidogenesis. Having demonstrated this PKA-MAPK/ERK axis as a critical conduit for skeletal regulation of steroidogenesis, we then defined its functional necessity. Although ERK has previously been shown to activate CREB in other cell types [20,21,22,23], its role in testosterone production remained unclear. Critically, we confirmed that ERK translocates to the nucleus and directly phosphorylates CREB. Inhibition of PKA, MEK, or ERK abolished both CREB phosphorylation and steroidogenesis, further elucidating the necessity of this integrated signaling network.

Protein phosphorylation at specific sites plays a pivotal role in cellular signal transduction by modulating protein activity and function. Dual phosphorylation of MEK at Ser218 and Ser222 enhances its catalytic activity, leading to efficient ERK activation [41,42]. Among these, Ser222 is recognized as a key activation site within the MAPK/ERK cascade [43,44]. Here, we found that exogenous ucOCN enhances MEK phosphorylation not only at the canonical activation sites (Ser218/Ser222) but also at Ser298, further potentiating ERK signaling. Within the MAPK signaling network, RAC activation involves its transition to a GTP-bound state (RAC-GTP), which facilitates the recruitment and activation of PAK. PAK subsequently phosphorylates MEK at Ser298 [40,45]. This phosphorylation stabilizes the Raf–MEK complex and enhances signaling efficiency along the MEK/ERK axis [46,47], contributing to MAPK-mediated regulation of cell proliferation, survival, and differentiation [48]. Thus, we propose that ucOCN binding to GPRC6A activates RAC, leading to PAK-mediated phosphorylation of MEK at Ser298 and subsequent ERK activation.

Moreover, using advanced CUT&Tag technology, we provide direct evidence that CREB binds to promoter regions of steroidogenic genes in response to ucOCN stimulation, thereby validating the long-standing hypothesis that CREB transcriptionally regulates testosterone synthesis. This binding was functionally correlated with enhanced gene expression and testosterone secretion.

In conclusion, ucOCN interacts with the membrane receptor GPRC6A in porcine Leydig cells, thereby activating the PKA-MAPK/ERK-CREB signaling axis that converges on the transcriptional activation of steroidogenic enzymes. Our study provides a fundamental molecular mechanism for future in vivo investigations of testosterone synthesis and spermatogenesis. These findings will significantly advance our understanding of skeletal-endocrine regulation on male reproduction and suggest ucOCN and its downstream effectors as potential targets for therapeutic intervention in hypogonadism and infertility. Furthermore, this study elucidates a new paradigm for exploring bone-derived hormones in reproductive physiology and provides a robust mechanistic foundation for future clinical applications.

## Data Availability

The data that support the findings of this study are available from the corresponding author upon reasonable request.

## Supplementary Materials

The following supporting information can be downloaded at: https://www.mdpi.com/article/10.3390/cells14241937/s1, Figure S1: High similarity of OCN between pigs and humans. Figure S2: Isolation and identification of porcine Leydig cells. Figure S3: CUT&TAG analysis demonstrating binding of p-CREB to the genes encoding four key enzymes required for testosterone synthesis (including HSD3B1). Figure S4: ucOCN exhibits no adverse effects on porcine Leydig cells. Figure S5: ucOCN exhibits no adverse effects on porcine vascular endothelial cells. Table S1: The antibodies. Table S2: The gene-specific primers used in RT-QPCR analyses.
