# Supplementary material for: ucOCN Promotes Testosterone Synthesis via the PKA-MAPK/ERK-CREB Signaling Pathway in Porcine Leydig Cells
Source: Cells. 2025 Dec 5;14(24):1937. doi: 10.3390/cells14241937 (PMC12730804; doi:10.3390/cells14241937)
Supplement: Supplementary file 1 [file cells-14-01937-s001.zip › cells-3996256-supplementary/Supplementary Materials/OCN-Supplementary Figure-cells.pdf]

A

Species	Human	Pig	Mouse
Human		84.49	68.63
Pig	86.00		70.23
Mouse	64.89	65.96	

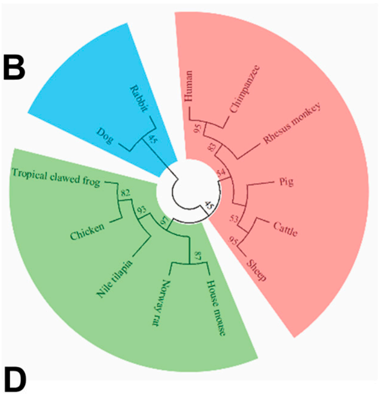

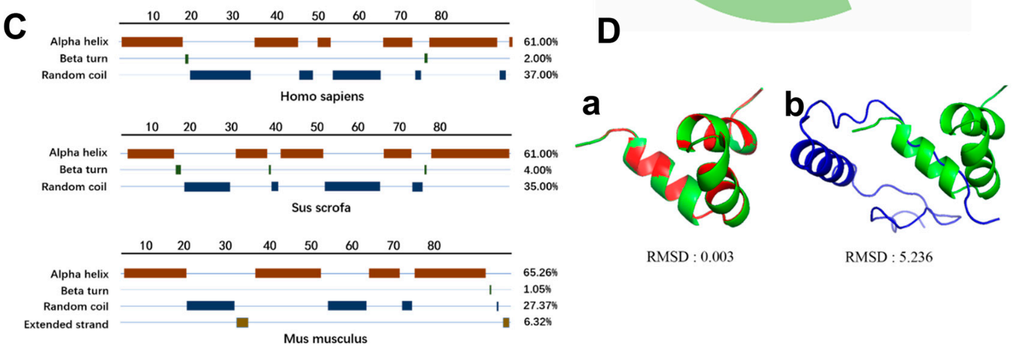

Figure S1 High similarity of OCN between pigs and humans. (A) Nucleotide and amino acid sequence identity alignment of OCN across pig, mouse, and human. (B) Phylogenetic tree analysis of OCN proteins from pig, mouse, and human. (C) Secondary structure prediction of OCN proteins from pig, mouse, and human. (D) Comparative analysis of OCN tertiary structures: a. Structural alignment of human (green) and pig (red) OCN tertiary prediction models; b. Structural alignment of human (green) and mouse (blue) OCN tertiary prediction models.

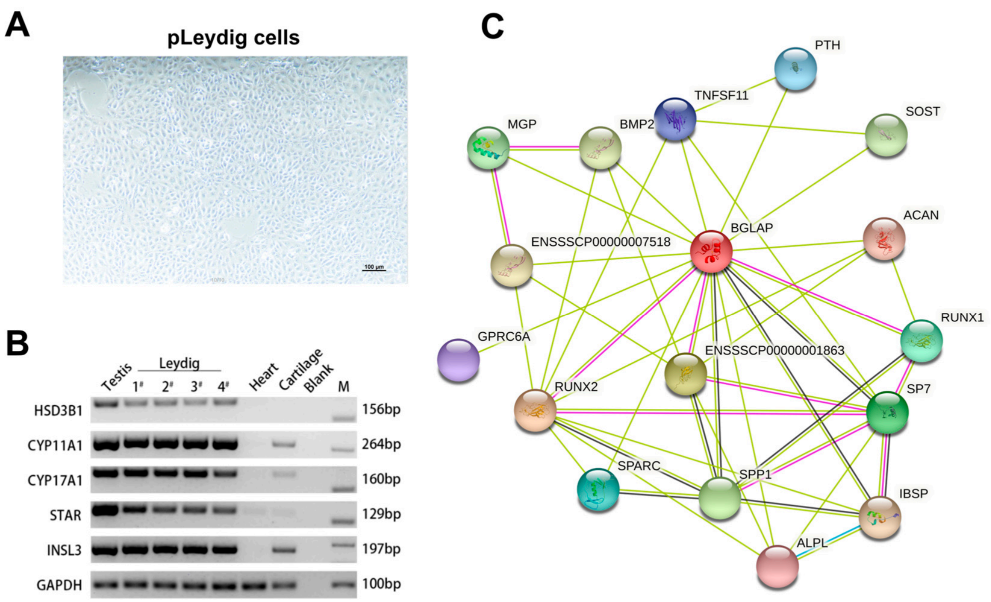

Figure S2 Isolation and identification of porcine Leydig cells. (A) Isolation of porcine Leydig cells from testis using Percoll gradient centrifugation. (B) RT-PCR detection of the expression of key enzyme genes required for testosterone synthesis (such as *HSD3B1*) and Leydig cell marker INSL3 in primary porcine Leydig cells. (C) Prediction of OCN-interacting proteins.

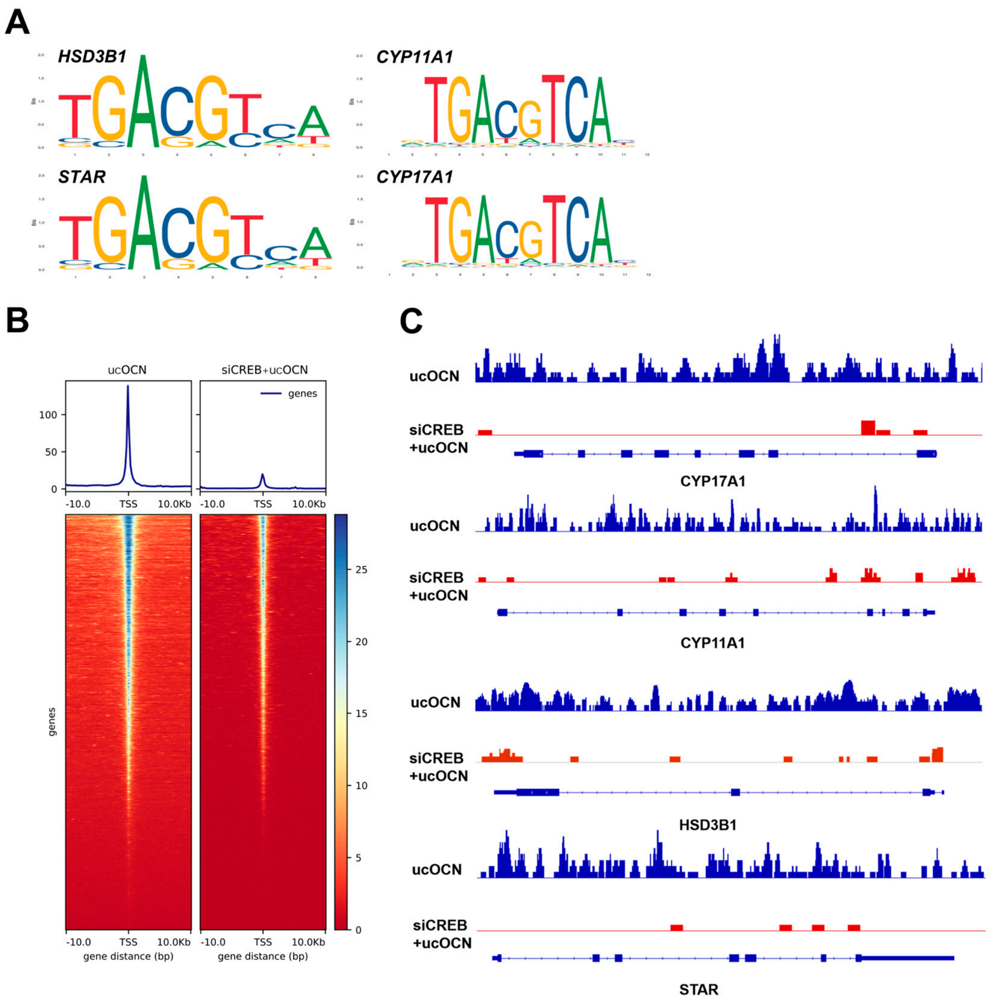

Figure S3 CUT&TAG analysis demonstrating binding of p-CREB to the genes encoding four key enzymes required for testosterone synthesis (including HSD3B1). (A) Prediction using the JASPAR database (jaspar.genereg.net/) that the transcription factor CREB binds to the promoter regions of testosterone-synthesizing key enzyme genes (*CYP11A1, CYP17A1, HSD3B1, STAR*) at the consensus site TGACGTCA, with the corresponding sequence logo shown. (B, C) CREB knockdown reduced its occupancy at the promoters of key steroidogenic genes (n =2 biological replicates).

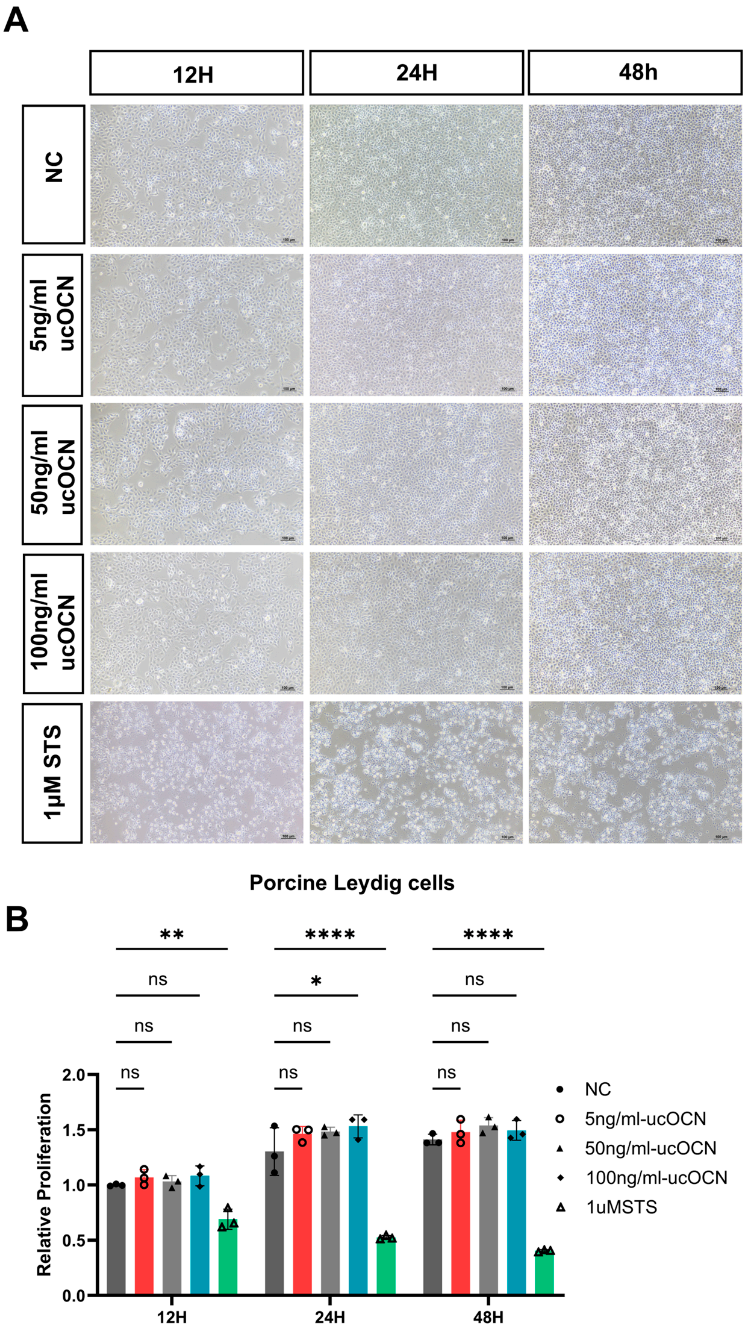

Figure S4 ucOCN exhibits no adverse effects on porcine Leydig cells. (A) Representative images of untreated porcine Leydig cells and cells treated with 5, 50, or 100 ng/mL ucOCN, or 1μM STS for 12, 24, and 48 h (Scale bars = 100 μm). (B) CCK-8 proliferation assay of untreated porcine Leydig cells and cells treated with 5, 50, or 100 ng/mL ucOCN, or 1μM STS for 12, 24, and 48 h (n = 3 biological replicates).

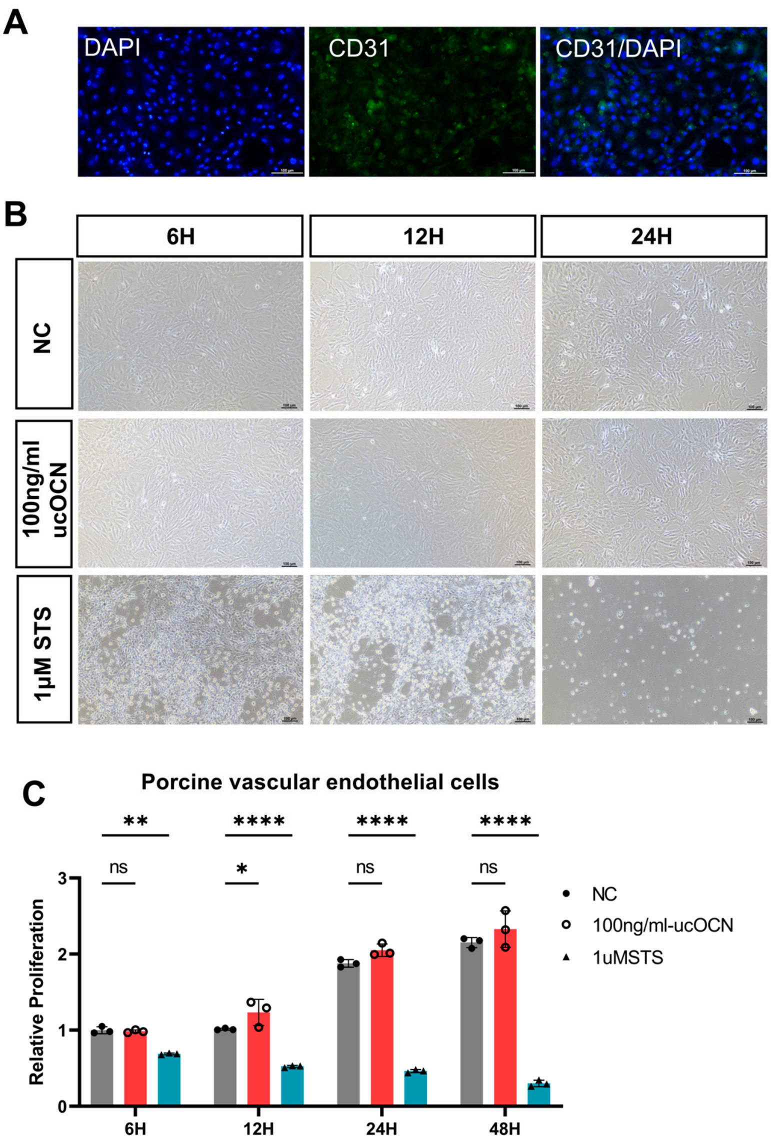

Figure S5 ucOCN exhibits no adverse effects on porcine vascular endothelial cells. (A) Immunofluorescence staining confirming expression of the endothelial-specific marker CD31 in primary porcine vascular endothelial cells (Scale bars = 100 μm). (B) Representative images of untreated porcine vascular endothelial cells and cells treated with 100 ng/mL ucOCN or 1μM STS for 6, 12, 24, and 48 h (Scale bars = 100 μm). (C) CCK-8 proliferation assay of untreated porcine vascular endothelial cells and cells treated with 100 ng/mL ucOCN or 1μM STS for 6, 12, 24, and 48 h (n = 3 biological replicates).
