# Supplementary material for: ucOCN Promotes Testosterone Synthesis via the PKA-MAPK/ERK-CREB Signaling Pathway in Porcine Leydig Cells
Source: Cells. 2025 Dec 5;14(24):1937. doi: 10.3390/cells14241937 (PMC12730804; doi:10.3390/cells14241937)
Supplement: Supplementary file 1 [file cells-14-01937-s001.zip › cells-3996256-supplementary/Supplementary Materials/Supplementary Table S1.pdf]

Antibody of WB	Company	Catalog Number	Actual Dilution Ratio
GAPDH	Abclonal	A19056	1:50000
p-ERK1/2	HUABIO	ET1610-13	1:2000
ERK1/2	HUABIO	ET1601-29	1:2000
p-MEK1/2 (S218 + S222)	HUABIO	ET1609-50	1:1000
p-MEK1/2 (S298)	HUABIO	ET1612-40	1:1000
p-PKA	HUABIO	HA721864	1:1000
PKA	HUABIO	HA723003	1:1000
STAR	Proteintech	12225-1-AP	1:1000
CYP17A1	Proteintech	14447-1-AP	1:1000
CYP11A1	Proteintech	13363-1-AP	1:1000
HSD3B1	HUABIO	HA500082	1:1000
GPRC6A	Abclonal	A8525	1:500
OCN	Proteintech	23418-1-AP	1:1000
H3	Proteintech	17168-1-AP	1:4000
CREB	Proteintech	12208-1-AP	1:4000
P-CREB	HUABIO	ET7107-93	1:1000
MEK1/2	HUABIO	ET1603-20	1:1000
Goat Anti-Rabbit IgG H&L (HRP)	Abclonal	AS014	1:10000
Goat Anti-Mouse IgG H&L (HRP)	Abclonal	AS003	1:10000
HRP Conjugated Rabbit IgG kappa light chain Mouse Antibody	HUABIO	M1208-2	1:5000

Antibody of IF	Company	Catalog Number	Actual Dilution Ratio
CYP17A1	Proteintech	14447-1-AP	1:50
CYP11A1	Proteintech	13363-1-AP	1:100
HSD3B1	Proteintech	HA500082	1:50
STAR	Proteintech	12225-1-AP	1:100
GPRC6A	HUABIO	ER63622	1:50
P-CREB	HUABIO	ET7107-93	1:50
p-ERK1/2	HUABIO	ET1610-13	1:100
iFluor™ 488 Conjugated Goat anti-mouse IgG	HUABIO	HA1125	1:500
iFluor™ 594 Conjugated Goat anti-rabbit IgG	HUABIO	HA1122	1:500
