# Supplementary material for: ucOCN Promotes Testosterone Synthesis via the PKA-MAPK/ERK-CREB Signaling Pathway in Porcine Leydig Cells
Source: Cells. 2025 Dec 5;14(24):1937. doi: 10.3390/cells14241937 (PMC12730804; doi:10.3390/cells14241937)
Supplement: Supplementary file 1 [file cells-14-01937-s001.zip › cells-3996256-supplementary/Supplementary Materials/Supplementary Table S2.pdf]

Table S2. The gene-specific primers used in RT-QPCR analyses.

	Sequence (5'-3')
GPRC6A-F	CAAACTCTTGCCATGATACAC
GPRC6A-R	TAAACTCCACGATTTCTCCA
HSD3B1-F	TCATCCACACTGCCTCTATC
HSD3B1-R	GTCCAGCCACCTCTATGC
STAR-F	ACCTCGTCCCCATTCTCCTG
STAR-R	GGTTCTCAGCTGGAAGACACT
CYP11A1-F	CACCCCATCTCCGTGACC
CYP11A1-R	GCATAGACGGCCACTTGTACC
CYP17A1-F	AAGACGAACGCAGAAAGTAAC
CYP17A1-R	GAGGATAGTGTAGCAGGAAGG
INSL3-F	GATCCAGAGGGGGATCCAGT
INSL3-R	CAAAGCCAAGGGTCCTCCAA
GAPDH-F	ACTCACTCTTCTACCTTTGATGCT
GAPDH-R	TGTTGCTGTAGCCAAATTCA
OCN-F	CGAGGTGGTGAAGAGACTCA
OCN-R	ATGCCATAGAAGCGCCGATAG
CREB-F	AACCAGCAGAGTGGAGATGC
CREB-R	CTGCTGGCATAGATACCTGGG
